# Six nations: a clinical scenario comparison of systems for prisoners with psychosis in Australia, Bolivia and four European nations

**DOI:** 10.1192/bji.2022.16

**Published:** 2023-02

**Authors:** Anne Aboaja, Prashant Pandurangi, Susana Almeida, Luca Castelletti, Guillermo Rivera-Arroyo, Annette Optiz-Welke, Justus Welke, Stephen Barlow

**Affiliations:** 1 PhD, MRCPsych, Consultant Forensic Psychiatrist, Forensic Service, Roseberry Park Hospital, Tees, Esk & Wear Valleys NHS Foundation Trust, Middlesborough, UK. Email: anne.aboaja@york.ac.uk; 2 FRCPsych, FRANZCP, Consultant Forensic Psychiatrist, Victorian Institute of Forensic Mental Health (Forensicare), Melbourne, Australia; 3 MD, Consultant Psychiatrist, Psychiatric and Mental Health Clinic, São João de Deus Prison Hospital, Lisbon, Portugal; 4 MD, Consultant Psychiatrist, Dipartimento Salute Mentale, AULSS 9, Verona, Italy; 5 MD, Professor of Psychopathology, Department of Psychology, Universidad Privada de Santa Cruz, Bolivia; 6 PhD, Consultant Forensic Psychiatrist, Institute of Forensic Psychiatry, Charité University Berlin, Germany; 7 MD, MSc, Epidemiologist, Institute of Forensic Psychiatry, Charité University Berlin, Germany; 8 FRCPsych, Consultant Forensic Psychiatrist, Nottinghamshire Healthcare NHS Foundation Trust, Rampton Hospital, Retford, UK

**Keywords:** forensic mental health services, prison, involuntary treatment, psychotic disorders, global inequalities

## Abstract

This paper compares across six nations the mental health systems available to prisoners with the highest acuity of psychosis and risk combined with the lowest level of insight into the need for treatment. Variations were observed within and between nations. Findings highlight the likely impact of factors such as mental health legislation and the prison mental health workforce on a nation's ability to deliver timely and effective treatment close to home for prisoners who lack capacity to consent to treatment for their severe mental illness. The potential benefits of addressing the resulting inequalities are noted.

## Background

At least 407 000 (3.7%) of the 11 million prisoners worldwide have a psychotic illness.^1,2^ The prevalence of psychosis among prisoners is higher in low- and middle-income nations than in high-income nations.^1,3^ Globally, there is high variability in the systems supporting the delivery of mental healthcare in the general population.^4^ A mental healthcare system comprises ‘all the organisations, people, and actions whose primary intent is to promote, restore or maintain [mental] health’.^5^ The system is therefore more than mental health facilities; it includes the workforce, medicines and relevant legislation.^6^

The aims of the paper are to (a) briefly describe the prison mental health systems that govern the care pathway of sentenced prisoners with psychosis in the six nations in which the authors work (Australia, Bolivia, England, Germany, Italy and Portugal) and (b) highlight the extent of any variation in the prison mental healthcare systems between these six nations, with a focus on Bolivia.

A clinical scenario of a prisoner with psychosis who refuses treatment and lacks capacity to consent to treatment was created and agreed (Box 1). Authors then provided nation-specific responses to the scenario, detailing the care pathway the prisoner was most likely to follow in practice within each nation's system. This was based on their knowledge of working as clinical and/or academic psychiatrists in forensic psychiatry. Using publicly available sources, the prison and mental health profile of included nations was compiled to provide context to the scenario responses (Table 1).

Box 1 Clinical scenario of a prisoner with psychosis

Mateo is a 24-year-old man in a closed prison serving a 3-year sentence for robbery. After serving 3 months of the sentence, he is observed by prison officers to be placing his cooked food in his shoes, wrapping toilet tissue around his face to cover his eyes and repeatedly asking officers ‘Why are you spying on me?’. Although he has a history of illicit substance misuse, his urine drug screen is negative. He is referred to the prison health centre. He is assessed by a psychiatrist who gives a working diagnosis of schizophrenia and prescribes an oral antipsychotic. Mateo refuses to take the medication. He believes it is poisonous and thinks prison staff are tampering with his food. He is heard shouting at night when alone in his cell, as if responding to hallucinations. Over the next few weeks his speech becomes incomprehensible and his self-care declines significantly. He appears to have lost weight. He threatens to punch anyone who enters his room. He attempts to set fire to his right hand. The psychiatrist working in the prison is called to review Mateo urgently. Mateo does not believe he is mentally unwell and continues to refuse medication. What are the lawful options for ensuring Mateo receives the necessary mental healthcare and that risks are adequately managed?

**Table 1:** Prison and mental health profile of the six nations

Nation	Income level (based on World Bank categories)	Prison population rate (prisoners per 100 000 population)	Prison occupancy level (number of prisoners/official prison capacity), %	Proportion of total health expenditure spent on mental health, %	Psychiatrists per 100 000 population, *n*
Australia	High	160	112.2	4.48	13.53
Bolivia	Lower middle	164	363.9	0.24	1.13
England	High	130	104.0	5.91	7.6
Germany	High	77	78.7	7.43	13.20
Italy	High	89	105.5	2.93	5.98
Portugal	High	111	87.6	5.72	11.95

Data collated from: Walmsley,^2^ the World Bank (www.worldbank.org/en/home) and the World Health Organization (www.who.int/).

## Nation-specific responses to the clinical scenario

### Australia

In Australia, each state has its own Mental Health Act.^7^ In Victoria, Mateo would already be in the prison's acute forensic mental health unit receiving 24-hour mental healthcare from a multidisciplinary team (MDT). However, under the Mental Health Act 2014 (Vic),^8^ in Victoria, in contrast to some other states in Australia, Mateo cannot receive pharmacological treatment involuntarily in prison. Since he is refusing treatment and his mental state has deteriorated with increased risks, he would be ‘certified’ under the Act for transfer from prison to a forensic hospital, where he can receive involuntary treatment. While awaiting transfer, he may be placed in an observation cell to manage suicide risk or moved to a non-health management unit for prisoners with violent behaviour. Once stabilised in hospital and no longer requiring involuntary treatment, Mateo would be transferred back to prison.

### Bolivia

There is no mental health law in Bolivia. Most prisoners have no access to mental health professionals and it is highly unlikely that Mateo would be reviewed by a psychiatrist or receive mental healthcare. However, if prescribed an antipsychotic by a visiting doctor, the medication would most likely be administered by a fellow inmate. Mateo would only receive involuntary treatment if displaying violence or other behaviours of concern. If he cannot access psychiatric treatment in the prison for his psychosis, he will remain untreated. As a result, he may lose the ability to pay his prison cell rent and subsequently be forced to sleep in the ‘free’ but crowded communal hall. Mateo may be placed alone in a prison ‘dungeon’ to manage the risk of harm to others.

### England

The Mental Health Act 1983, which governs the involuntary treatment of people with a mental disorder, cannot be used to enforce treatment in a prison against a person's will.^9^ A visiting or full-time psychiatrist working as part of the prison mental health team will refer Mateo to a secure psychiatric hospital. If it is agreed that he should be transferred to hospital for treatment, two doctors will submit separate medical reports to the Ministry of Justice. The Secretary of State for Justice will issue a warrant for Mateo's transfer to hospital under section 47 of the Mental Health Act. This process should take no more than 14 days, but in practice, the waiting times for secure hospital transfer are often substantially longer.^10^ While waiting to transfer to a secure hospital, which may be a considerable distance from the prison and his home, Mateo will continue to receive care and support from the prison mental health team.

### Germany

In Germany, the medical treatment of prisoners is regulated by federal laws of the 16 federal states (*Strafvollzugsgesetze*). Where the mental state of a prisoner is associated with increased risk of violence towards others, suicide or self-injury, the prisoner can be detained in a specially secured cell and, if necessary, physically restrained. Involuntary medical treatment cannot be administered without a court decision. A judgment is usually made within 3–6 weeks or, if urgent, 1 week. While awaiting a judgment, all efforts will be made to persuade Mateo to accept medication. If, after a decision of the court, involuntary treatment is initiated, it may be continued in a prison health unit until Mateo regains the capacity to consent and can then decide for himself whether to accept further medical treatment.

### Italy

In an Italian prison, an ordinary correctional wing would not manage disturbed behaviour and the prison psychiatric observation ward does not admit inmates with severe mental illness. A prison psychiatrist would refer Mateo to an *Articolazione Tutela Salute Mentale in carcere* (ATSM), a regional correctional service with a full MDT providing in-patient psychiatric care for prisoners. Admission to an ATSM is managed by local correctional psychiatric services and requires the primary evaluation of a magistrate's court. However, involuntary treatment cannot be given in any prison setting. Involuntary treatment is governed by a *Trattamento Sanitario Obbligatorio* (involuntary treatment order) under Law number 833/1978,^11^ which states that pharmacological treatment can only be lawfully administered in an acute psychiatric hospital. Therefore, given his refusal of medication, Mateo would be transferred from the ATSM to an acute psychiatric hospital to receive involuntary treatment for as long as clinically indicated, before returning to prison.

### Portugal

National mental health legislation does not apply in prisons in Portugal. Instead, prison mental healthcare is governed by prison legislation.^12,13^ Mateo can only receive involuntary medical treatment in prison if there is a danger to his life or a serious threat to his health and he lacks capacity to consent to medication. Treatment can be administered only in the Prison Hospital, where psychiatrists are on-call 24 h a day. Mateo would transfer to the only Prison Hospital in the country. Unlike national mental health law, prison legislation states that Mateo cannot receive involuntary treatment after he recovers from the acute psychotic episode. The psychiatrist cannot continue medication without Mateo's consent and, if well, Mateo would return to prison. There is no legal framework for prisoners to enforce medication to prevent relapse (as is available to the non-prisoner population through the national Mental Health Law) and this results in a ‘revolving door’ pattern of repeated admissions to the Prison Hospital.

## Comparison of systems for prisoners with severe mental illness

### Mental health legislation

With the exception of Bolivia, all six reviewed countries have legislation governing the mental healthcare of prisoners and stipulating conditions (criteria, setting, duration) for applying involuntary treatment. This takes the form of either general mental health legislation that applies to prisoners (Australia, Italy, England) or prison legislation that focuses on mental health (Germany, Portugal).

### Mental health specialists in prison

Although specialist mental health assessment and interventions appropriate to the level of illness acuity and need is recommended^14^ and is available in some nations, in Bolivia the majority of prisoners with a psychotic illness do not have access to a specialist mental health professional but would more probably receive mental healthcare from a professional who has not undergone specialist mental health training, sometimes with the informal support of a peer worker. This is not surprising, given the low number of psychiatrists per 100 000 population, the high prison population and the elevated occupancy level (Table 1), which mean that there are likely to be fewer psychiatrists available to meet the mental health needs in prisons. However, in some nations with a larger mental health budget and a higher number of psychiatrists per capita, prisoners can also access a full mental health MDT (Australia, Portugal, Germany).

### Criteria for involuntary treatment

Medication refusal and/or lack of capacity to consent to treatment are the criteria used in most of the nations for involuntary treatment. In some countries involuntary treatment is unlawful once capacity is regained (Germany) or when the acute phase of illness has passed (Portugal), whereas in other countries involuntary treatment may continue until the patient is discharged from hospital (Australia, England, Italy).

### Setting for involuntary treatment

In some nations involuntary treatment can be administered in prison (Bolivia, Germany and Portugal), although in the last two nations, the inmate must first be transferred to a specified prison mental health unit. In contrast, in Italy, England and some states of Australia, involuntary treatment for mental disorder cannot be enforced in prison and can only be administered in a hospital.

### Timescales for involuntary treatment – the patient experience

The length of time between when a doctor recognises that a patient refuses or lacks capacity to take medication and when involuntary treatment can be lawfully commenced contributes to the duration of untreated psychosis (DUP), and a long DUP is associated with poor health outcomes and recidivism.^15,16^ This waiting time varies between nations. In Bolivia, where involuntary treatment is not governed by legislation, medication can be commenced immediately. However, in Germany and England, where permission for involuntary treatment must first be sought from higher authorities and arrangements made for transfer to either a prison hospital or psychiatric hospital, the patient experiences a delay of weeks before commencing treatment.

## Discussion

### System variability and health inequalities for prisoners with psychosis

We have described the systems for delivering mental healthcare to prisoners with psychosis in the six nations reviewed and highlighted considerable variability. The differences between systems and prisoner experience arise from variations in legislation and resources and can lead to significant health inequalities for prisoners with psychosis in the six nations compared, particularly in Bolivia.

Potential inequalities in mental healthcare between the prisoner and general population that arise from this variability have been identified: legislation applicable to the prisoner population that does not support relapse prevention (Portugal); resource limitations hindering access to mental health professionals in prisons (Bolivia) (Table 1); systemic delays occurring in a prisoner's access to an environment in which involuntary treatment can be administered (England); and the requirement of multiple referrals and transfers to access involuntary treatment (Italy). Additional within-nation mental health inequalities may affect prisoners where legislation differs across the nation (Australia).

Between-nation mental health inequalities have been highlighted where a prisoner with acute psychosis in some nations will have access to a full multidisciplinary mental health team in prison (Australia), whereas a prisoner in a less well-resourced nation may not see a mental health specialist (Bolivia). Additional inequalities exist between healthcare systems in which the prisoner must be transferred to an institution at increased distance from home (or the prison) to receive involuntary treatment (Portugal, England) and healthcare systems that could offer involuntary treatment closer to the prison in which the sentence is being served (Bolivia). Inequity between nations arises when a prisoner with capacity who refuses treatment might be left untreated in one nation (Germany) but administered treatment against their will in another (England).

### Contextualisation and implications of findings

Our findings of mental healthcare inequalities between prisoners and non-prisoners, as well as between prisoners in different nations, are consistent with findings of a study comparing pathways to mental healthcare for prisoners in 24 European nations.^17^ A high level of legislative variability between nations was also reported in a multi-continent, scenario-based comparison of systems for prisoners with mental disorders, although the study focused on pre-trial prisoners and court disposals.^18^ Wide variation in forensic in-patient service provision among 17 European nations was observed and tentatively linked to variations in country income level and the proportion of the overall budget allocated to healthcare.^19^

Our comparison not only replicates earlier findings that legislation and resources (e.g. workforce) are key factors contributing to differences in prison mental health systems between nations,^18,20^ but explores the impact of these systemic differences on the care available to sentenced prisoners with psychosis, and considers them within a health inequality framework. For example, we found that legislation, or the lack thereof, can determine whether, when, where and for how long involuntary treatment can be administered to prisoners, and it can contribute to delays in accessing treatment for patients refusing or lacking capacity. This paper contributes to the current ethical debate on balancing human rights to health, to life and to not be tortured in the context of people with a mental disorder who might receive involuntary treatment.^21^ Furthermore, we extend thinking in the field of global prison mental health by highlighting the impact that diverse understandings and practices of involuntary treatment may have on the timely and effective treatment of sentenced prisoners with psychosis and, consequently, their prognosis.

### Limitations

Although the use of a clinical scenario to elicit pathway-focused responses from practising clinicians proved effective in also highlighting the patient experience of delays in different systems, there are two notable limitations in our comparison. First, the absence of low-income countries and the inclusion of only one middle-income nation alongside five high-income nations means that the stark differences between Bolivia and the other nations must be interpreted cautiously. Second, although the male gender assigned to the scenario character reflects the high proportion of males in prisons worldwide, the provision of and access to mental health services may be unequal between the two genders. Similarly, there may also be differences according to prisoner ethnicity and age but the scenario did not allow these themes to be identified through an intersectional lens and explored in the context of health inequalities. The evidence that combinations of gender, age and ethnicity can affect an individual's experience of the criminal justice^22^ and mental health systems^23–25^ strengthens the recommendation that health inequalities be examined through an intersectional lens in the investigation of prison mental health systems.^25^

## Conclusions

The variation in the responses to the clinical scenario highlights a lack of consensus across nations regarding involuntary treatment for prisoners with psychosis. A global shared understanding of ethical best practice regarding this matter could influence legislation and would mark an important step towards reducing some of the inequalities identified. Given the improved health outcomes and reduced recidivism associated with effective treatment of psychosis in prisoners,^16^ it is important that prison mental health systems worldwide are evaluated and compared. Studies that identify the barriers to timely, effective treatment of psychosis and evaluate the systems and pathways that might reduce health inequalities and protect the human rights of prisoners with psychosis can inform the development of legislation, policy, guidance, workforce and resources sufficient to meet the mental health needs of the growing prison population worldwide.

## Data Availability

Data availability is not applicable to this article as no new data were created or analysed in this study.
