# Six Nations: a clinical scenario comparison of systems for prisoners with psychosis in Australia, Bolivia and four European nations – ERRATUM

**DOI:** 10.1192/bji.2023.10

**Published:** 2023-08

**Authors:** Anne Aboaja, Prashant Pandurangi, Susana Almeida, Luca Castelletti, Guillermo Rivera-Arroyo, Annette Opitz-Welke, Justus Welke, Stephen Barlow

Keywords: forensic mental health services; prison; involuntary treatment; psychotic disorders; global inequalities; erratum

This article was originally published with a spelling error in the name of author Annette Opitz-Welke. This has now been corrected and this erratum published.
